# Forgetting rates of gist and peripheral episodic details in prose recall

**DOI:** 10.3758/s13421-022-01310-5

**Published:** 2022-04-13

**Authors:** Riccardo Sacripante, Robert H. Logie, Alan Baddeley, Sergio Della Sala

**Affiliations:** 1 Human Cognitive Neuroscience, Department of Psychology, University of Edinburgh, Edinburgh, EH8 9JZ UK; 2 Department of Psychology, University of York, Heslington, York, UK

**Keywords:** Episodic memory, Long-term forgetting, Gist, Repeated retrieval, Prose recall

## Abstract

In a seminal study, Slamecka and McElree showed that the degree of initial learning of verbal material affected the intercepts but not the slopes of forgetting curves. However, more recent work has reported that memories for central events (gist) and memory for secondary details (peripheral) were forgotten at different rates over periods of days, with gist memory retained more consistently over time than details. The present experiments aimed to investigate whether qualitatively different types of memory scoring (gist vs. peripheral) are forgotten at different rates in prose recall. In three experiments, 232 participants listened to two prose narratives and were subsequently asked to freely recall the stories. In the first two experiments participants were tested repeatedly after days and a month, while in the third experiment they were tested only after a month to control for repeated retrieval. Memory for gist was higher than for peripheral details, which were forgotten at a faster rate over a month, with or without the presence of intermediate recall. Moreover, repeated retrieval had a significant benefit on both memory for gist and peripheral details. We conclude that the different nature of gist and peripheral details leads to a differential forgetting in prose free recall, while repeated retrieval does not have a differential effect on the retention of these different episodic details.

## Introduction

In a seminal paper, Slamecka and McElree (1983) investigated the role of the degree of learning in normal forgetting using lists of words from different categories (Experiment 1), lists of unrelated word-pairs (Experiment 2), and sentences with both verbatim and gist memory (Experiment 3), at three time intervals (soon after presentation, 1 day and 5 days). At encoding, participants were presented with different numbers of study trials to control for the degree of initial performance. They showed that the number of study trials, hence the degree of initial performance, affected the intercepts but not the slopes of the forgetting curves. It was argued that forgetting of verbal material appeared to be independent of the degree of initial performance (i.e., learning).

Similarly, studies assessing forgetting rates of meaningful prose passages or narratives observed that the slopes of forgetting curves did not change as a function of the degree of initial learning over retention intervals of 2 days (Gilbert, 1957). With a forced-choice test, Christiaansen (1980) observed a hierarchy of retention of different types of information of a prose passage (main character, paragraph, sentence gist, sentence wording) with no differential forgetting rates across them. More recently, Rivera-Lares et al. (2022) presented lists of unrelated sentences for delayed cued recall and found no difference in forgetting rates as a function of initial memory performance.

On the other hand, research assessing retrieval and coherence of mnemonic representations has reported a fragmentation of the memory traces, with some aspects being forgotten more rapidly than others (Brady et al., 2013; Joensen et al., 2020; Lifanov et al., 2021). When assessing forgetting of dependencies among three elements regarding a given event (person, location, and object), Joensen et al. (2020) found that dependencies among these three elements were stable across time intervals (immediate, 12 h and 1 week). Thus, central events were either completely preserved or completely lost in a so-called *all-or-none* fashion. Nevertheless, they also noted a fragmentation of the memory trace, as some aspects (e.g., contextual information or peripheral details) tend to decline differentially, some more gradually than others (see also Horner & Burgess, 2013, 2014).

Accordingly, memory for different types of events would be expected to decline at different, negatively accelerated rates (Conway et al., 1991; Sekeres et al., 2016; Thorndyke, 1977). Memory for central events (gist) of prose material would be retained more robustly over time (Brainerd & Reyna, 2002; Heuer & Reisberg, 1990; Koutstaal, 2006) than details or secondary (peripheral) information, which is generally forgotten more rapidly (Bartlett, 1932; Brainerd & Reyna, 2002; Sachs, 1967; Sekeres et al., 2016; Tulving, 1972). More recent research assessing forgetting of different memory details also observed a faster rate of forgetting for peripheral details (Winocur et al., 2010; Winocur & Moscovitch, 2011). According to the Trace Transformation Theory (TTT; Moscovitch & Gilboa, 2021; Sekeres et al., 2018), detailed episodic memories are transformed into memories lacking details while still retaining the gist features of the events. This theory has gained support from brain-imaging (Bonasia et al., 2018; St-Laurent et al., 2016; Sekeres et al., 2020), cross-sectional (St-Laurent et al., 2014) and longitudinal behavioural studies (Brady et al., 2013; Lifanov et al., 2021; Sekeres et al., 2016). Given the theoretical predictions of TTT, it would therefore be expected that memory for details (peripheral) would be forgotten significantly more than memory for central events (gist) over long-term intervals.

These predictions are also consistent with the Fuzzy Trace Theory (FTT; Brainerd & Reyna, 2002), which postulates a distinction between different levels of representation, namely gist and verbatim. More specifically, Brainerd and Reyna (2005) argued that the evaluation of forgetting curves should allow for a distinction between gist and verbatim memory. These authors hypothesized that forgetting would differ among memory scoring type, with verbatim memory dropping to floor while gist memory remains more accessible and stable over time (Brainerd & Reyna, 2005; for a recent review, see Helm & Reyna, in press).

However, only a few studies have so far assessed the forgetting rates of gist and peripheral memory in complex material such as prose free recall over long-term memory intervals. Heuer and Reisberg (1990) used a 2-week incidental recall task for central elements and peripheral details of a narrative related to an emotional event. In this study, emotional arousal promoted memory for both central and peripheral information of an event. However, when participants were explicitly instructed to closely attend to the event, memory for gist was better than for peripheral details. Other studies assessing memory and emotion (Burke et al., 1992; Christianson & Loftus, 1991; Reisberg & Heuer, 1992) reported higher memory scores for the gist and lower accuracy for visual details at time intervals up to 2 weeks.

In a further study from Sekeres and co-workers (2016), participants were asked to watch a series of film clips and to freely recall the content of the story (gist) and any perceptual detail (peripheral) at three retrieval sessions (10 min, 3 days and 7 days). To avoid repeated testing, the clips were divided into three series, one each to be tested at each retrieval session. Sekeres et al. (2016) demonstrated that peripheral details of event-based memories were forgotten more rapidly than gist events using intervals up to 1 week (Experiment 1). Therefore, participants showed a greater time-dependent loss of peripheral details, as observed by the significant interaction between time interval and memory detail. In our own experiments, we aimed to assess the forgetting rates of memory for gist and peripheral details in prose recall at delays of up to a month.

Another issue derives from practice effects that typically occur in repeated retrieval designs (Roediger & Karpicke, 2006; Roediger & Butler, 2011). In their Experiment 3, Sekeres et al. (2016) repeatedly tested memory for the same gist and peripheral memories (soon after presentation and at 1 day, 3 days and 7 days) and demonstrated that repeated retrieval promoted the recollection of both types of memory detail and, most importantly, prevented the loss of peripheral details over time intervals of up to a week. Indeed, with this testing protocol, retention interval and memory detail did not interact.

A series of recent studies (Baddeley et al., 2014; Baddeley et al., 2019) employed a novel prose recall test, the Crimes Test, which is not demanding in terms of initial learning, and allows for free and cued recall of different subsamples of questions at different delays. Baddeley et al. (2019) compared the performance of participants tested repeatedly (immediately, 1 day, 1 week and 1 month, i.e., interpolated testing condition) and a group of participants tested on different subsamples of the material at each delay with a group tested with no intervening recall. Participants tested only after 1 month showed greater forgetting. These authors proposed that repeated testing promoted the activation of both the features directly tested together with the priming of other non-assessed features resulting in their slower rate of forgetting. Baddeley et al. (2021) went on to show that this priming effect resulted from using integrated episodes and was not found with lists of independent words or scenes.

Such studies have, therefore, consistently demonstrated that delayed memory performance on prose recall is relatively well maintained when testing the same material on multiple occasions. Stamate et al. (2020), using similar prose material (fables), recently observed that this also holds true for patients with Alzheimer’s disease when tested at the same intervals as Baddeley et al. (2019). Hence, in the current study we assessed the forgetting rates of memory for gist and peripheral details by controlling for repeated testing.

Furthermore, it is still unclear whether initial levels of performance lead to differential forgetting rates of memory for gist and peripheral details. Despite previous research using verbal and prose material (Gilbert, 1957; Slamecka & McElree, 1983) reporting that the slopes of forgetting curves did not change as a function of initial degree of learning, recent research has demonstrated that the speed at which people learn information predicts memory retention up to several days (McDermott & Zerr, 2019; Zerr et al., 2018; for a review, see McDermott, 2021) suggesting a link between learning and forgetting. In their study, Zerr et al. (2018) assessed forgetting rates in participants who studied Lithuanian-English word pairs and were subsequently tested on immediate cued-recall. Corrective feedback was applied so non-recalled items were tested until each pair was recalled correctly. Faster learners outperformed slower learners from initial testing, and they showed better retention on the final test. This prolonged retention among faster learners was also observed at longer time intervals (Nelson et al., 2016; Zerr, 2017) and extended to visuospatial material (Zerr et al., 2021). These recent findings reiterate the advantage that higher learners hold over time, yet they do not seem to contradict the notion of parallel slopes in forgetting.

The main question remains whether or not there is an interaction between initial levels of performance and the rate of forgetting across the time intervals at which memory retention is assessed. Given the contrasting results in previous studies as to whether or not levels of initial performance have an impact on forgetting rates, the present experiments aim to investigate the forgetting rates of qualitatively different types of memory scoring (gist vs. peripheral), over delays ranging from 1 day up to 1 month, by controlling for repeated retrieval.

Differences in forgetting rates could emerge when the study material is tested at longer time intervals (e.g., a month) rather than intervals of days (see Slamecka & McElree, 1983), meaning that such differences could be time-dependent (Sekeres et al., 2016; see also Sadeh & Pertzov, 2020). Recent research conducted by Fisher and Radvansky (2018) has accordingly noted a shift in the pattern of forgetting prior to and after 7 days, with markedly increased forgetting after a week for both word-lists and prose material. Fisher and Radvanksy (2018) also argued that much of the published research on forgetting does not allow for an assessment of changes in forgetting patterns over time, as data usually are collected before or after the 7-day interval (see also Radvansky et al., 2022).

In the first two experiments reported here, we investigated whether forgetting rate depends on the type of memory scoring (gist vs. peripheral) by adapting the experimental design devised by Slamecka and McElree (1983). The paradigm was designed to investigate whether differences in memory for gist and for peripheral details immediately after encoding would predict rates of forgetting for each.

It is possible that the repeated retrieval of the study material at different retention intervals in Experiment 1 and Experiment 2 could have benefitted the recollection of memory for gist more than for peripheral details (Jansari et al., 2010). Alternatively, repeated retrieval of a story might enhance the retention of memory for both gist and peripheral events (Carpenter et al., 2008; Sekeres et al., 2016, 2020; Yonelinas, 2002). To address the issue of the possible impact of repeated retrievals (for a review, see Roediger & Butler, 2011), a third experiment was conducted in which participants’ retrieval of gist and peripheral memory was assessed only immediately after presentation and after a month’s delay.

## Experiment 1

This first experiment examined the differences in the strength of qualitatively distinct episodic memory scoring type (gist vs. peripheral) over long-term intervals (from a few days to a month). Forgetting of distinct types of memory scoring was assessed through free verbal recall of two brief prose narratives that were auditorily presented during an initial session.

Previous studies reported differential forgetting of central and secondary elements in episodic memory (Conway et al., 1991; Sekeres et al., 2016; Thorndyke, 1977). Here we aimed to assess whether this finding replicates with the longer time interval of a month.

### Methods

#### Participants

A total of 60 young adults (42 women and 18 men) aged 18–34 years (*M* = 22.11, *SD* = 3.89) were recruited from the general population and were tested in a lab of the University of Edinburgh. Their total years of formal education ranged from 12 to 18 years (*M* = 15.65, *SD* = 1.80). All the participants were native English speakers and none suffered from hearing loss by self-report.

All participants signed an informed consent and were given a small honorarium.

#### Material

Two prose narratives (see Table 1) were selected from previous studies (St-Laurent et al., 2014; Sekeres et al., 2016). These were assigned a fixed balanced score for gist and peripheral memory, following the procedures derived from previous studies (Sacripante et al., 2019; Sekeres et al., 2016; St-Laurent et al., 2011, 2014). Each story was structured with five sentences and concerned a single episode. The total number of words included in the stories was 73 for Story A and 59 for Story B. The stories lasted approximately 26 s each and they were narrated by a male (Story A) and a female voice (Story B) with a standard English accent.

**Table 1:** Illustration of the narratives presented to the participants at the immediate time interval

**Story A: Crashing the Bicycle**	
A boy and his dad are riding a bike, with the boy sitting on the handlebars.	
They are going down a hill. The father squeezes the brakes to slow them down.	
He realizes that the brakes are broken. They both scream as the bike accelerates.	
The dad tries to brake with his shoes, without success.	
They hit a tree on the side of the road at full speed and fall off the bike.	
**Gist scores (5)**	
Man and boy on bicycle going down a hill	
Man finds out brakes don’t work	
Man tries to slow down	
They crash into tree	
They fall off of bike	
**Peripheral scores (5)**	
Boy sitting on the handlebars	
Both scream	
With his shoes	
On a side of the road	
At full speed	
**Story B: Woman Squeezing Food**	
An elderly woman is in a food store, handling a peach.	
She squeezes the fruit so hard that it bursts and splatters her in the face.	
The man behind the counter gives her an angry look.	
Embarrassed, she vanishes down one of the aisles, while he follows her.	
She starts squeezing a soft cheese with her thumbs, looking delighted.	
**Gist scores (7)**	
Woman in grocery store	
Woman is squeezing a peach	
Woman squeezes so hard juice squirts out	
Cashier is angry/surprised	
Woman runs away	
Cashier follows her	
Woman squeezes cheese	
**Peripheral scores (7)**	
So hard	
In the face	
Behind the counter	
Down one of the aisles	
Soft (cheese)	
With her thumbs	
Looking delighted	

The two stories together had a maximum score of 12 for both gist and peripheral memory. Following previous research (Sacripante et al., 2019; Sekeres et al., 2016; St-Laurent et al., 2014), gist memory items were defined as a precise recall of “what happened” during the passage, in relation to the event, the people involved, interaction and actions. Peripheral memory items were defined as a precise recall of specific details, involving appearance of people and objects (“looking delighted”), relative position of characters and objects (“the man behind the counter”), position of main character in relation to objects (“the boy sitting on the handlebars”), facial or vocal expressions (“they both scream”), motion qualifiers and sound (“at full speed”). For instance, for Story A, remembering “a father and his son are riding on a bike” would be considered as a central event (gist), while the fact that “the son was sitting on the handlebars” would be considered as a secondary information or detail (peripheral). In Story B, remembering that a “woman started to squeeze a cheese” would be considered as gist, while the fact that the cheese was “soft” would be considered as a peripheral information.

In our scoring procedure partial credits were not included, so if participants recalled “a man and boy on a bicycle going down a hill” they would be given the same score of 1 for gist as in “man and boy on a bike” (consistent with the scoring protocol reproduced in Table 1). As memory scores were assigned leniently (see *Procedure* section), other similar versions of the event were given a gist score (e.g., “father and son are on a bike ride” or “a son and his dad are out on a bicycle”).

The classification of gist memory events was based on previous research studies that initially used this prose material (Sekeres et al., 2016; St-Laurent et al., 2014), while peripheral memory events were initially classified by one of the researchers (R.S.).

Prior to data collection, the classification and distinction of text as gist or peripheral was validated with a pilot study involving six independent judges (all PhD students at the University of Edinburgh and all fluent English speakers). Three of them were asked to read the stories and to provide a list of events that should be classed as central (gist) or secondary details (peripheral). The other three judges were asked whether they agreed with the proposed classification. The feedback provided by the six judges helped us to select and modify the list of gist and peripheral items to reach a consensus among experimenters and judges. The final list used in the study is shown in Table 1.

Inter-rater reliability of the scores was analysed by comparing a subset of 60 scores (15 participants × 2 stories × 2 memory types) given by the experimenter (R.S.) to those of a second rater not involved in the study. The Krippendorf’s alpha was 0.96, which indicates a good agreement among the two raters.

#### Procedure

**Fig. 1:**
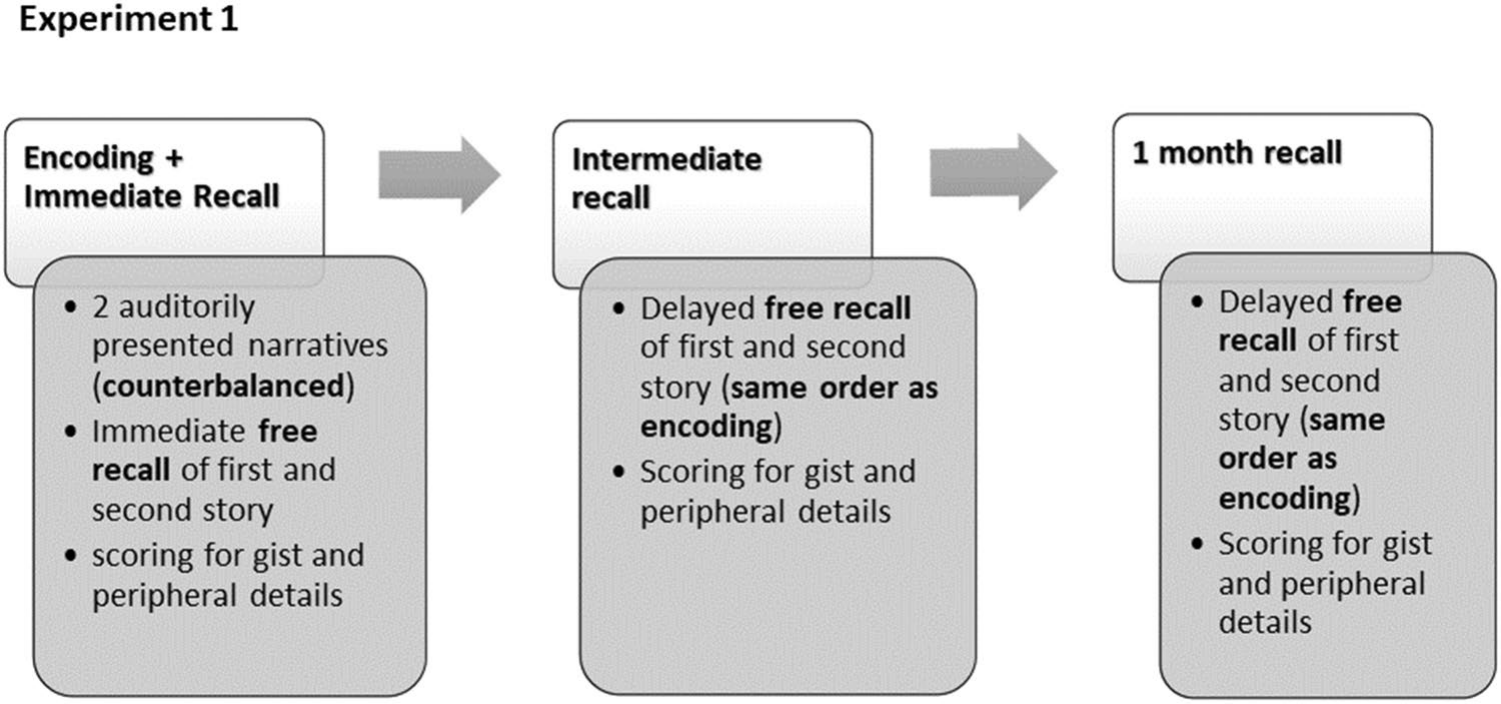
Illustration of the experimental design of Experiment 1

The procedure of Experiment 1 is summed up in Fig. 1. Participants were asked to listen carefully to two narratives through headphones; they were made aware that they would be tested later on what they could remember about the stories. The order of the narratives and the gender of the narrator were counterbalanced to avoid order effects. The narratives were presented through a pair of headphones attached to a computer screen.

After listening to the stories (one after the other), participants were asked to immediately recall what they could remember of each story in the order presented in one single recording. This was assessed by two separate recording sessions in which the participants were asked to perform a free recall of the first and the second story without any specific instruction regarding central or peripheral events. The order of recall for gist and peripheral information was not counterbalanced as participants provided their recollection for both memory for gist and peripheral details in one single recording of the story. This recall procedure differed from the one from Sekeres et al. (2016), who instead prompted recall for gist and for peripheral memory in two separate recording sessions. A maximum of 1 min was allowed for the free recall of each of the stories. The experiment was carried out using E-Prime2 (version 2.0.10.242, E-Studio, Psychology Software Tools Inc.).

After attending the first session in the lab, participants were tested after 1 day, 3 days or 5 days to ascertain any possible performance difference at shorter intervals, then they were tested again after a month. Participants were assigned to the testing delay according to their individual availability to come back to the lab after 1 day, 3 days or 5 days. There was no reason to assume that this resulted in any systematic bias in the allocation of participants to each delay group. A total of nine participants were not available for follow-up testing after the first session, hence 51 participants were assessed on the longer retention intervals. Among these, 18 were tested after 1 day, 17 after 3 days, and 16 after 5 days.

In the second testing session, following the delay, participants physically came back to the lab to freely recall the stories in the lab, and their recall was audio-recorded. The scores for both gist and peripheral memory were assigned by the experimenter (R.S.) according to a pre-set grid, listening to the recordings obtained from the participants.

After a period of a month, all participants were contacted again by phone by the experimenter for unexpected follow-up testing. Delayed testing over the phone has been a longstanding method to collect data on memory performance (Baddeley et al., 2014, 2019; Houston, 1969; Runquist, 1983), and it has been found not to be detrimental to performance (Allen et al., 2019). All 51 participants agreed to be retested, after they provided their contact details during their last in-person session. On this occasion, participants were asked to freely recall the two stories and their scores were assigned by the experimenter using a tick-list form that included all the original prose passages and the memory scores for gist and peripheral memory (see Table 1). Participants were not recorded at 1 month as these were surprise phone calls, which happened unexpectedly. The ethical approval for the study required that participants sign a consent form about being recorded during the two sessions that took place in the lab; we did not have ethical approval for consent for recording to be obtained over the phone.

A lenient (i.e., not strictly verbatim) scoring criterion was applied, as previously done by Slamecka and McElree (1983) in their experiments. False memories or items recalled from one narrative while attempting to retrieve another (intrusions) were recorded.

Participants who scored at floor for either gist or peripheral memory at immediate recall were excluded from further analyses. No participants were excluded due to a floor score for gist memory, while one participant was excluded due a floor score for peripheral memory.

### Results

In this experiment, the sample size was selected by prioritizing a balanced number of participants for each delay group (1, 3 and 5 days), for a total of 20 participants for each group. No statistical difference was observed across the three intermediate time-recall groups in relation to memory scores on the overall test performance, as evidenced by a non-significant main effect on the between-subjects variable of time recall (*p* = 0.72). Therefore, participants from 1-, 3- and 5-day intermediate testing groups were assumed to be matched. The collapse of the delay groups into a single group and the exclusion of the between-subjects variable of delay group allowed us to increase the power of the sample size with a purely within-subjects design.

A 2 × 3 repeated-measures design was used, with memory scoring type (gist vs. peripheral) and recall time (immediate vs. intermediate vs. month) as within-subjects variables. Memory score was the dependent variable.

Given the non-normal distribution of the data, analyses included generalized linear mixed-effects modelling to test the forgetting rates (slopes) of gist and peripheral memory decay over the three recall intervals. Statistical analysis was computed with R (version 4.0.3).

**Fig. 2:**
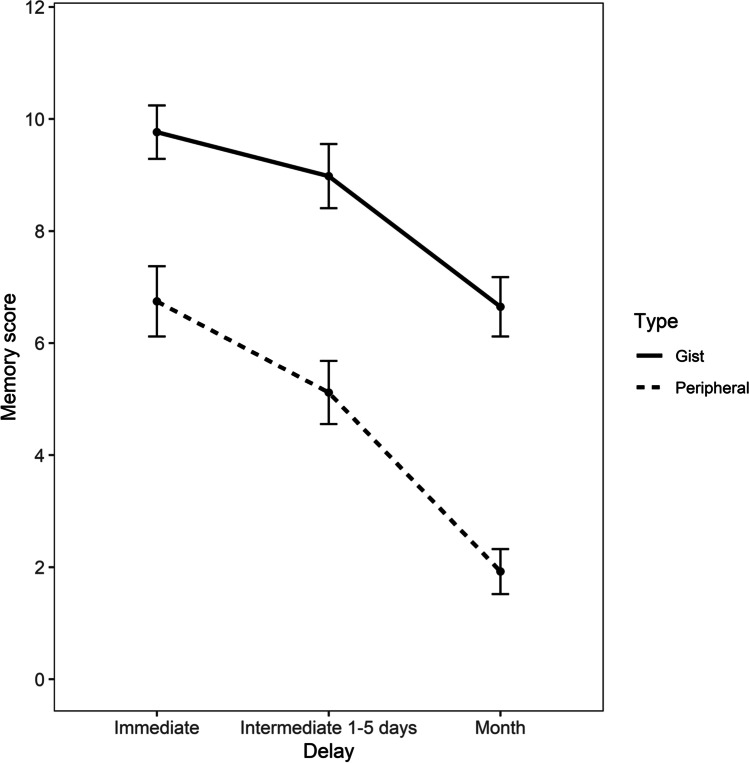
Mean gist and peripheral memory scores with confidence intervals (95% CIs) at immediate, intermediate and 1-month delays in Experiment 1

Memory scores were analysed with a generalized linear mixed model fit by maximum likelihood as implemented by the lme4 package (Bates et al., 2015) in R. In this model, the outcome variable represented the number of correct responses out of 12 questions, which was the maximum score possible (Score, 12- Scores). Correct responses were defined by the number of units of information (i.e., events) that were correctly recalled for each story. As the data did not follow a normal distribution, they were instead modelled using the binomial distribution (family = binomial). The fixed effects considered the interaction between memory scoring type (gist and peripheral) and recall (immediate, intermediate and month) with a random intercept for participants (cbind(Score, 12 -Score) ~ Type*Recall + (1|ID)). The intercept of this model was gist memory scores at immediate recall. Results are reported in Fig. 2. Descriptive data are provided in Table 2.

**Table 2:** Descriptive statistics of gist and peripheral memory scores at the three time intervals (Immediate, Intermediate and Month) for Experiment 1, including 95% confidence intervals (CIs) around the mean

Delay	Gist				Peripheral			
	Mean	SD	SE	95% CI	Mean	SD	SE	95% CI
Immediate	9.76	1.69	0.23	9.28–10.24	6.74	2.22	0.31	6.11–7.37
1–5 days	8.98	2.03	0.28	8.40–9.55	5.11	2.00	0.28	4.55–5.68
Month	6.64	1.88	0.26	6.11–7.17	1.92	1.42	0.19	1.52–2.32

The within-subjects factor of memory scoring predicted performance at immediate recall, as gist memory scores were significantly higher than peripheral memory scores, *b* = -1.31, *SE* = 0.13, *z* = -9.60, *p* <.001, *d* = 0.72.

The within-subjects factor of recall affected gist memory scores, as the differences in memory scores between immediate and intermediate intervals, *b* = -0.40, *SE* = 0.14, *z* = - 2.84, *p* <.01, *d* = 0.22, and between immediate and month intervals, *b*= -1.35, *SE* = 0.13, *z* = -9.87, p < .001, *d* = 0.74, were both significant.

In relation to the interaction between memory scoring and recall, peripheral memory scores did not decrease significantly more than gist memory scores from immediate to intermediate interval, *b* = - 0.18, *SE* = 0.18, *z* = -0.97, *p* = 0.33, *d* = 0.09, meaning that they were forgotten at the same rate. However, peripheral memory scores decreased significantly more than gist from immediate to month interval, *b* = -0.68, *SE* = 0.19, *z* = - 3.49, *p* < .001, *d* = 0.37. This means that peripheral memory scores were forgotten at a faster rate than gist memory scores after a month.

To better explore this significant interaction, post hoc comparisons with a Bonferroni correction were carried out as implemented by the package emmeans (Lenth, 2021). Post hoc comparisons revealed that peripheral memory scores also decreased significantly more than gist from intermediate to month interval, *b* = - 0.50, *SE* = 0.19, *z* = - 2.62, *p* < .05, *d* = 0.27.

As can be seen from Fig. 2, peripheral memory after a month approached floor performance (13.7%). The same statistical approach was employed after excluding all those participants who performed at floor at any time interval. The sample size decreased from 51 to 44 participants and the results were the same as when all participants were included. After excluding floor performance, the analysis of the interaction between memory scoring and recall showed that peripheral memory scores did not decrease significantly more than gist memory scores from immediate to intermediate interval, *b* = - 0.23, *SE* = 0.20, *z* = -1.14, *p* = 0.25, *d* = 0.12, while they decreased significantly more than gist from immediate to month interval, *b* = - 0.61, *SE* = 0.20, *z* = -2.93, *p* < .01, d = 0.33. Therefore, peripheral memory scores were forgotten at a faster rate than gist memory scores after a month even without the presence of floor performance.

Eighteen participants reported items not presented in the original narratives (i.e., false memories), for a total of 28 instances – six at immediate recall, 18 at intermediate recall and four after 1 month. Twenty-five instances of false memories were related to gist memory events (four at immediate recall, 17 at intermediate recall and four after 1 month), while the remaining three concerned peripheral memory events (two at immediate recall and one at intermediate recall). In this sample, only one participant recalled a peripheral memory item from one narrative while recalling another (i.e., intrusion) at intermediate recall, and no participants made multiple intrusions.

### Discussion

The present study assessed the forgetting rates of memory for gist and peripheral details of two prose narratives over time delays up to a month.

As expected, the type of memory scoring predicted the initial level of performance (Brainerd & Reyna, 2002). That is, gist memory scores were higher than peripheral memory scores. Also, both gist and peripheral memory scores declined across the three time periods (immediate, 1–5 days, and 1 month). In relation to peripheral memory, participants seemed to forget secondary details after a month at a faster, negatively accelerated rate.

This study has some limitations. Firstly, the extent to which participants could expect or anticipate the delayed recall of the stories was not controlled. Despite previous research evidence ruling out an effect of expectation on memory performance (Houston, 1969; Runquist, 1983), active rehearsal could have biased the outcome over different retention intervals if participants guessed that they might be asked for delayed recall.

Another issue relates to the lack of consistency regarding the testing context. While all participants had to attend the first and the second session in a laboratory setting, all the participants were tested remotely by phone call at a month’s recall. It could be argued that a difference in testing context is not optimal.

Finally, the delay for intermediate testing varied between 1 and 5 days across participants. Although the analysis showed the performance did not differ as a result of that variability, it is possible that it added “noise” variance to the data across participants that might have made the intermediate test session insensitive and underestimated any forgetting that occurred.

## Experiment 2

To address the limitations listed above, a follow-up study was conducted by keeping the intermediate time interval at 3 days for all the participants with a third session after 1 month. Participants were not made aware of a follow-up session, rather they were just contacted by phone without notice. Similarly, the testing context was kept consistent across testing sessions, with a first face-to-face session followed by two remote phone follow-up sessions.

### Methods

#### Participants

A total of 82 young adults (53 women and 29 men) aged 18–35 years (*M* = 23.39, *SD* = 3.96) were recruited from the general population.^1^ Their total years of formal education ranged from 13 to 18 years (*M* = 16.24, *SD* = 1.52). All the participants were native English speakers, and none suffered from hearing loss by self-report. None had taken part in Experiment 1.

All participants signed an informed consent and were given a small honorarium.

#### Material

The prose passages included in this study were the same used in Experiment 1 (Table 1).

#### Procedure

Participants were exposed to the stories with the same procedure as for Experiment 1, with only two follow-up sessions that took place exactly after 3 days and a month. Follow-up testing always occurred over the phone and three participants dropped out as they could not be reached.

### Results

A 2 × 3 repeated-measures design was employed, with memory scoring (gist and peripheral memory) and time interval (immediate, 3 days and 1 month) as within-subjects variable and memory score as outcome variable.

**Fig. 3:**
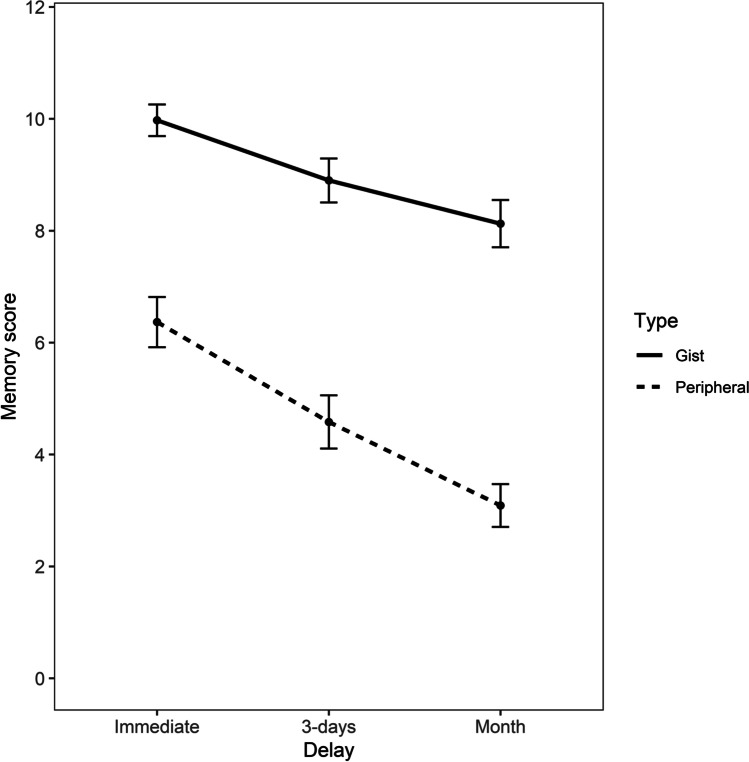
Mean gist and peripheral memory scores with confidence intervals (95% CIs) at immediate, 3-day and 1-month delays in Experiment 2

As in Experiment 1, statistical analysis was carried out with R and it included generalized linear mixed-effects modelling fit by maximum likelihood to test the forgetting rates (slopes) of gist and peripheral memory decay over the two recall intervals (see *Results*, Experiment 1). The parameters and the package (lme4) used to implement this model did not change from the ones used in Experiment 1 (cbind(Score, 12 -Score) ~ Type*Recall + (1|ID)) and the data were modelled according to a binomial family distribution (family = binomial), as they were not normally distributed. Results are reported in Fig. 3. Descriptive data are provided in Table 3.

**Table 3:** Descriptive statistics of gist and peripheral memory scores at the three time intervals (Immediate, Intermediate and Month) for Experiment 2, including 95% confidence intervals (CIs) around the mean

Delay	Gist				Peripheral			
	Mean	SD	SE	95% CI	Mean	SD	SE	95% CI
Immediate	9.97	1.26	0.14	9.69–10.25	6.36	2.00	0.22	5.91–6.81
3 days	8.89	1.75	0.19	8.50–9.29	4.58	2.12	0.23	4.10–5.05
Month	8.12	1.88	0.21	7.70–8.54	3.08	1.71	0.19	2.70–3.47

The within-subjects factor of memory scoring type predicted performance at immediate recall, as gist memory scores were significantly higher than peripheral memory scores, *b* = -1.55, *SE* = 0.11, *z* = -13.93, *p* < .001, *d* = 0.85.

The within-subjects factor of recall delay predicted gist memory scores, as the differences in memory scores between immediate and 3-day interval, *b* = -0.56, *SE* = 0.11, *z* = -4.84, *p* <.001, *d* = 0.30, and between immediate and month interval, *b* = -0.89, *SE* = 0.11, *z* = -7.87, *p* <.001, *d* = 0.49, were both significant.

In relation to the interaction between memory scoring type and recall, peripheral memory scores did not decrease significantly more than gist memory scores from immediate to 3-day interval, *b* = -0.07, *SE* = 0.15, *z* = -0.50, *p* = 0.61, *d* = 0.03, while they decreased significantly more than gist from immediate to month interval, *b* = -0.35, *SE* = 0.15, *z* = -2.32, *p* = 0.01, *d* = 0.19. Again, this suggests that peripheral memory scores were forgotten at a faster rate than gist memory after a month.

However, post hoc comparisons with Bonferroni correction revealed that peripheral memory scores did not decrease significantly more than gist from the 3-day to the month interval, *b* = -0.27, *SE* = 0.14, *z* = - 0.62, *p* = 0.17, *d* = 0.14. This lack of significance could be due to a lower rate of floor scores for peripheral memory at month recall observed in Experiment 2 (3.8%) as compared to Experiment 1 (13.7%).

As performed in Experiment 1, the same statistical analyses were carried out after excluding all those participants who performed at floor at any time interval. The sample size decreased from 79 to 76 participants and the results were same as when all participants were included. The analysis of the interaction between memory scoring and recall showed that peripheral memory did not decrease significantly more than gist memory from immediate to 3-day interval, *b* = - 0.08, *SE* = 0.15, *z* = -0.55, *p* = 0.58, *d* = 0.04, while it decreased significantly more than gist from immediate to month interval, *b* = - 0.34, *SE* = 0.15, *z* = -2.25, *p* = 0.02, *d* = 0.18.

In this sample, 27 participants reported items not presented in the original narratives (i.e., false memories), for a total of 38 instances, 18 after 3 days and 20 after a month. Twenty-nine cases of false memories were related to gist memory events (13 at intermediate recall and 16 after 1 month), while the remaining nine concerned peripheral memory events (five at intermediate recall and four after 1 month). No participant recalled an item from one narrative while recalling another (i.e., intrusion).

### Discussion

This second experiment aimed to address some methodological issues of Experiment 1, by keeping a fixed intermediate time interval at 3 days and not making participants aware of the follow-up testing sessions. Also, the follow-up testing sessions were all carried out by telephone. With these modifications to the procedure, results from this experiment confirmed that memory for peripheral details was forgotten at a faster rate after a month when compared to memory for central, gist-based events. However, in both Experiment 1 and Experiment 2 the same material was tested at the intermediate delay and the 1-month delay. It is possible that this repeated retrieval might have provided more of a benefit to memory for gist than memory for peripheral details, leading to a steeper forgetting slope for the latter (Jansari et al., 2010; Roediger & Karpicke, 2006).

Experiment 3 aimed to address this issue by testing each participant only immediately after hearing the stories and then after 1 month.

## Experiment 3

To evaluate whether repeated retrieval influenced the patterns of forgetting in Experiment 1 and Experiment 2, a third experiment was conducted on a sample of younger adults without any testing at an intermediate delay.

### Methods

#### Participants

A total of 90 young adults (65 women and 25 men) aged 18–33 years (*M* = 21.63, *SD* = 2.97) were recruited from the general population.^2^ The total years of formal education ranged from 11 to 18 years (*M* = 15.94, *SD* = 1.79). All the participants were native English speakers, and none suffered from hearing loss by self-report. None had taken part in Experiments 1 or 2.

All participants signed an informed consent and were given a small honorarium.

#### Material

The prose passages included in this study were the same as those used in Experiment 1 and Experiment 2 (see Table 1).

#### Procedure

**Fig. 4:**
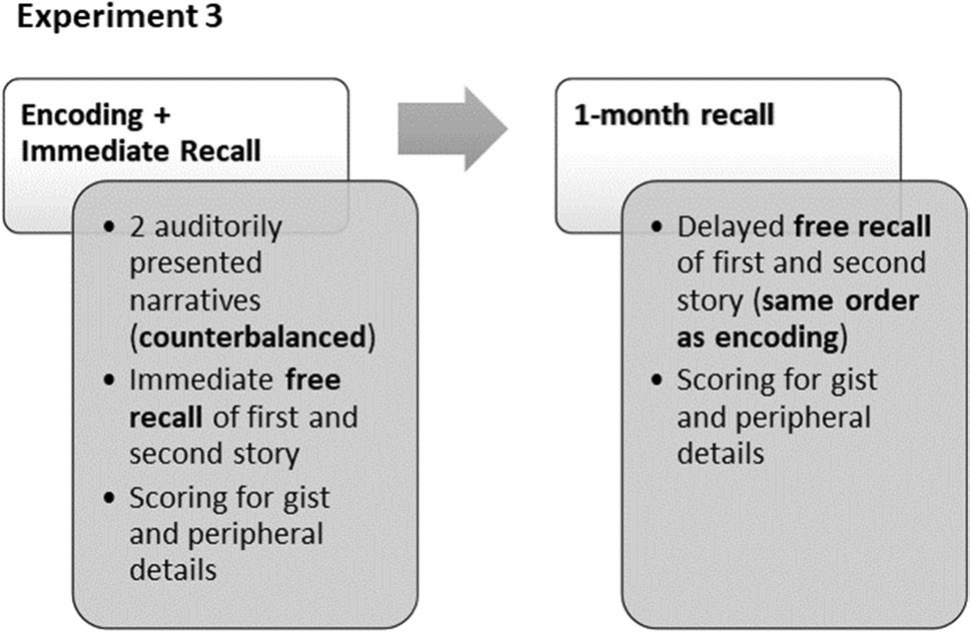
Illustration of the experimental procedure of Experiment 3

The procedure for Experiment 3 is shown in Fig. 4. Participants were exposed to the stories with the same procedure and apparatus as in the experimental procedure of Experiment 1, with only one follow-up session exactly after a month and always carried out over the phone. Three participants could not be contacted by telephone after the 1-month delay.

Memory scores were assigned according to the same procedure as in Experiment 1. No participant was excluded due to floor scores at immediate recall.

### Results

A 2 × 2 repeated-measures design was employed, with memory scoring type (gist vs. peripheral memory) and time interval (immediate vs. 1 month) as within-subjects variable and memory score as outcome variable.

**Fig. 5:**
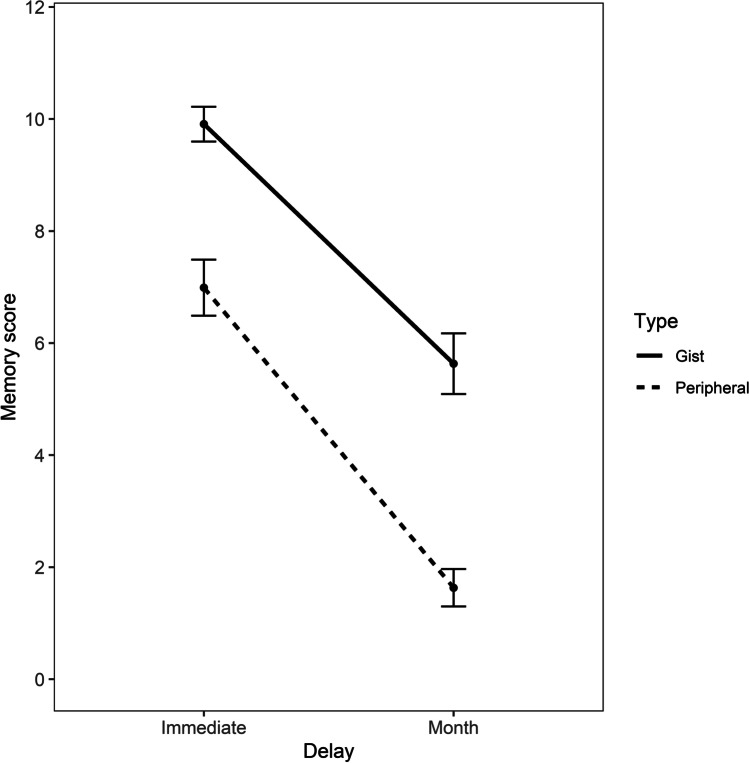
Mean gist and peripheral memory scores with confidence intervals (95% CIs) at immediate and 1-month delays in Experiment 3

As in Experiment 1 and Experiment 2, statistical analysis was carried with R and it included generalized linear mixed effects modelling fit by maximum likelihood to test the forgetting rates (slopes) of gist and peripheral forgetting at immediate testing and after 1 month. This model was implemented with the same parameters and package (lme4) used in Experiment 1 and Experiment 2 (cbind(Score, 12 -Score) ~ Type*Recall + (1|ID)) and the data were modelled according to a binomial family distribution (family = binomial), as they did not follow a normal distribution. For this model the variable Recall had only two levels (immediate, 1 month). Results are provided in Fig. 5. Descriptive data are provided in Table 4.

**Table 4:** Descriptive statistics of gist and peripheral memory scores at the two time intervals (Immediate and Month) for Experiment 3, including 95% confidence intervals (CIs) around the mean

Delay	Gist				Peripheral			
	Mean	SD	SE	95% CI	Mean	SD	SE	95% CI
Immediate	9.90	1.45	0.15	9.59–10.21	6.98	2.35	0.25	6.48–7.49
Month	5.63	2.53	0.27	5.09–6.17	1.63	1.56	0.16	1.29–1.96

The within-subjects factor of memory scoring predicted performance at immediate recall, as gist memory scores were significantly higher than peripheral memory scores, *b* = -1.30, *SE* = 0.10, *z* = -12.22, *p* <.001, *d* = 0.71.

The within-subjects factor of recall delay predicted gist memory scores, as the differences in memory scores between immediate and month intervals, *b*= - 1.79, *SE* = 0.10, *z* = - 16.80, *p* < .001, *d* = 0.98, were highly significant.

In relation to the interaction between memory scoring type and recall, peripheral memory scores decreased significantly more than gist from immediate to month interval, *b* = - 0.53, *SE* = 0.15, *z* = - 3.45, *p* < .001, *d* = 0.29. Therefore, peripheral memory scores were forgotten at a faster rate than gist memory scores after a month.

This finding was also replicated after excluding those participants who performed at floor at any time interval, with the sample size decreasing from 87 to 63 participants. The analysis of the interaction between memory scoring and recall showed that peripheral memory decreased significantly more than gist from immediate to a month interval, *b* = -0.42, *SE* = 0.17, *z* = -2.46, *p* = 0.01, *d* = 0.23.

In this sample, 20 participants reported items not presented in the original narratives (i.e., false memories), for a total of 26 instances, two at immediate and 24 after a month. Twenty-five cases of false memories were related to gist memory events (one at immediate recall and 24 after 1 month), while the remaining one concerned a peripheral memory event after 1 month. One participant recalled a gist memory item from one narrative while recalling another (i.e., intrusions).

To explore whether forgetting slopes of gist and peripheral memory were differentially affected by repeated testing, data from Experiment 2 (repeated retrieval) at 1-month delay and Experiment 3 (single testing) were compared by using the same statistical approach. Although this post hoc comparison was not formally part of the initial design, it was carried out as a further exploration of the overall pattern of data and as such is relevant to its broad interpretation. Also, the participants were different in each experiment, and there is no reason to believe that allocation to each experiment was non-random.

**Fig. 6:**
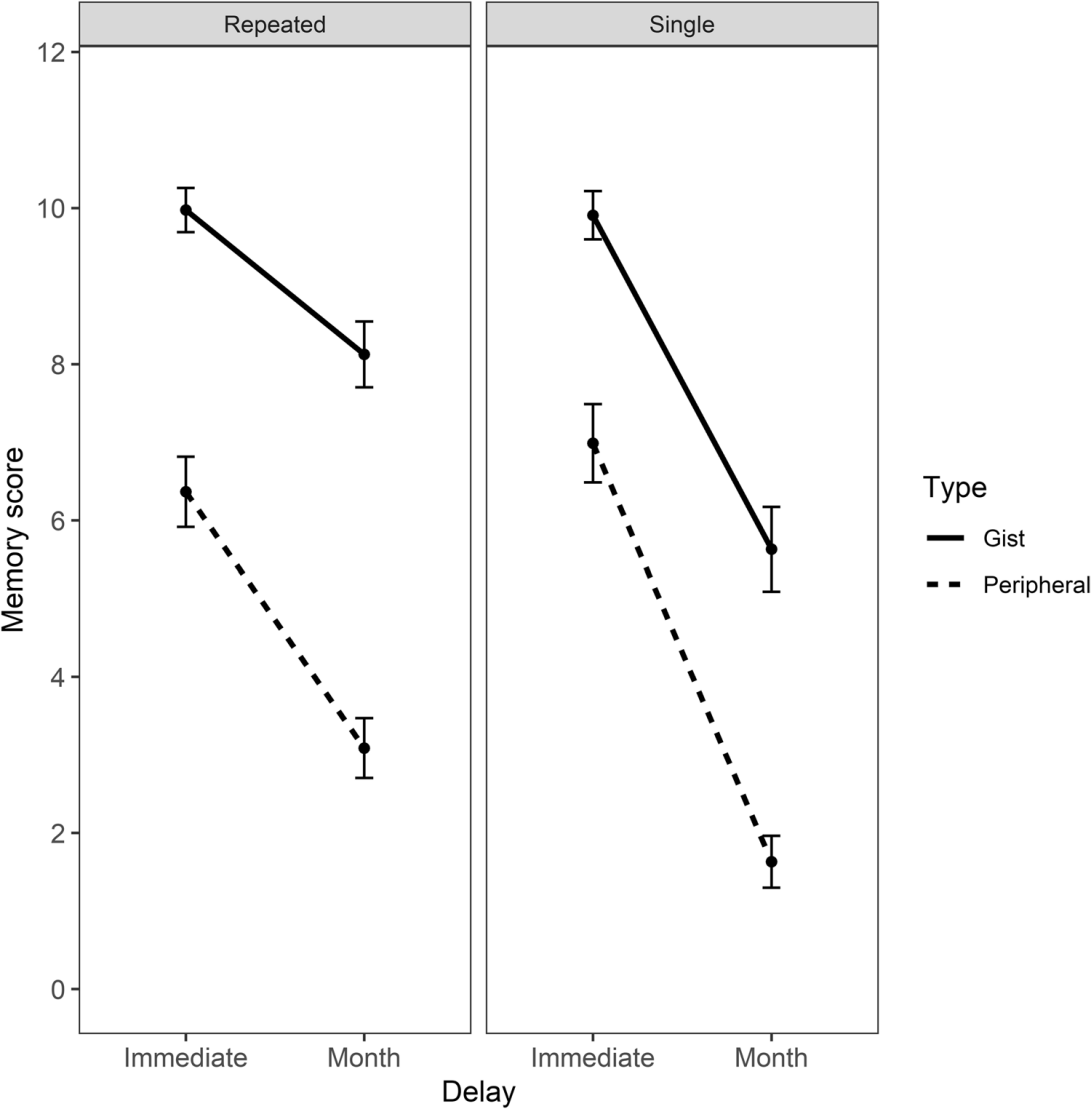
Mean gist and peripheral memory scores with confidence intervals (95% CIs) at immediate and 1-month delays, divided by repeated (Experiment 2) and single (Experiment 3) testing condition

Since the three-way interaction (Testing*Type*Recall) was not significant (*p* = 0.48), the model was simplified by specifying the interaction terms (Group + Type + Recall + Group:Type + Type:Recall + Group:Recall) and by adding a random intercept for participants (1|ID). The comparison between the two fitted models was performed with the anova() function and the models did not statistically differ (*p* = 0.41), meaning that they were equally parsimonious. Repeated testing resulted in higher scores for both gist and peripheral memory after a month compared to single testing, *b* = -0.98, *SE* = 0.10, *z* = -9.00, *p* < .001, *d* = 0.54 (see Fig. 6).

### Discussion

A greater forgetting for peripheral details was again observed even when the intermediate time point was excluded. These findings confirm that forgetting rates of qualitatively different types of memory scoring as a function of time do not result in parallel curves even after controlling for repeated retrieval at the intermediate time point. Therefore, the faster rate of forgetting in peripheral details that we observed in Experiments 1 and 2 was not influenced by repeated retrieval.

Repeated retrieval had a beneficial effect on retention of both gist and peripheral memory. This finding is consistent with the notion that repeated retrieval promotes retention and reactivation of both gist and peripheral elements of an episode (Sekeres et al., 2016, 2020). Indeed, repeated retrieval is linked to increased recollection (Carpenter et al., 2008; Yonelinas, 2002) and better accessibility of memory for gist and peripheral details for subsequent retrieval (Roediger & Butler, 2011).

## General discussion

The present series of experiments aimed at assessing whether forgetting rate depends on the nature of the memory scoring type and whether or not forgetting is influenced by repeated testing.

The methodology was inspired by the experimental design devised by Slamecka and McElree (1983). Participants were verbally presented with two brief prose passages and asked to perform a free verbal recall on both passages immediately and after a few days and after a month. At each time interval, all the participants were assigned a gist and a peripheral memory score based on their verbal recollection of the events from both stories.

Across three experiments, the type of memory scoring predicted the initial level of performance, as gist memory scores were generally higher than peripheral memory scores. This finding indicated that information is encoded and processed differently (Craik & Lockhart, 1972), with the central elements of a story being more salient than secondary details in verbal memory recollection (Christiaansen et al., 1978; Conway et al., 1991; Dooling & Christiaansen, 1977; Thorndyke, 1977).

Both gist and peripheral memory scores decreased across time intervals in all three experiments, with (Experiment 1 and Experiment 2) or without (Experiment 3) an intermediate time recall.

Across the three experiments, secondary details were forgotten at a faster, negatively accelerated rate compared to central events. The same faster, negatively accelerated forgetting of peripheral details was demonstrated even without the presence of an intermediate time interval between immediate and 1 month’s recall (Experiment 3).

To investigate whether the difference in forgetting rate between the two types of memory scoring was due to a differential impact of repeated retrieval, direct comparisons between participants from Experiment 2 and Experiment 3 revealed that repeated testing benefitted the recollection of both central events and secondary details.

Crucially, our findings may seem to be at odds with Slamecka and McElree’s (1983), as they do not show parallel forgetting rates regardless of initial level of performance. To resolve this apparent inconsistency, we propose that long-term forgetting could be accounted for by (at least) two processes: one that is time-based and, as demonstrated by Slamecka and McElree (1983), results in parallel slopes, and another that is material-based (see also Sekeres et al., 2016) resulting in diverging slopes with details being forgotten faster than gist.

The diverging forgetting slopes observed in our study could be due to a gradual erosion of episodic memory details over time, whereby different types of memory scoring (i.e., gist vs. peripheral) differ in their resistance to such erosion. This is also in line with the notion that forgetting has an adaptive role, as, for older memories (e.g., after a month), participants tend to forget secondary details and retain a generalised recollection of the event (Hardt et al., 2013; Moscovitch & Gilboa, 2021; Sadeh & Pertzov, 2020; Sekeres et al., 2016; Sekeres et al., 2018).

Furthermore, our experiments employed longer time intervals (i.e., a month) as compared to the ones adopted by Slamecka and McElree (1983). It is likely that differences in forgetting the study material might be time-dependent (Sekeres et al., 2016; see also Sadeh & Pertzov, 2020), as they could emerge at longer time intervals (a month) rather than at “shorter” intervals of days.

In our experiments, the significant differences in forgetting rates of gist and peripheral memory after a month could be explained by the notion that peripheral memory details may be particularly sensitive to time-dependent forgetting (Sadeh & Pertzov, 2020) and therefore become gradually less retrievable at longer time intervals. Our findings are also consistent with Fisher and Radvansky (2018), who observed that retention of a prose narrative was stable until 7 days, while they reported a marked change in the pattern of forgetting after this time interval (see also Radvansky et al., 2022). Thus, the length of time intervals might have played an influential role on the time-dependent forgetting of peripheral details reported in our experiments.

To resolve this issue, future research should, on the one hand, verify whether differences in the forgetting rate of gist and peripheral memory can also be observed at shorter time intervals. On the other hand, the experimental paradigm on initial degree of learning employed by Slamecka and McElree (1983) should also be replicated with time intervals beyond a week.

It is relevant to note that cueing a specific feature of an integrated prose involving crimes (Baddeley et al., 2014) or door scenes (Baddeley et al., 2019) as well as fables (Stamate et al., 2020) activates other related features within that specific event. Such associative boosts could be explained by a process of either strengthening or reactivation of an existing memory representation (Baddeley et al., 2021; Sekeres et al., 2016, 2020). In our experiments, the presence of an intermediate recall (i.e., intermediate testing) in Experiment 2 offered such reactivation for both gist and peripheral events.

Taken together, the outcome of our experiments shows that the different nature of gist and peripheral details plays a crucial role in forgetting and long-term memory retention in the context of prose free recall. Moreover, as expected, forgetting is influenced by repeated testing, which proved beneficial for the recollection of both central and secondary events.

## Footnotes

1 For Experiment 2, a larger sample size was included, based on a priori power analysis carried out on G*Power 3.1 (Faul et al., 2009), which indicated a minimum number of 60 participants, assuming a medium effect of 0.25, error probability at 0.05 and power at 0.99.

2 For Experiment 3, a larger sample size was again included, based on a priori power analysis carried out on G*Power 3.1 (Faul et al., 2009), which indicated a minimum number of 76 participants, assuming a medium effect of 0.25, error probability at 0.05 and power at 0.99.
